# Gluten-Free Diet: From Development to Assessment of a Check-List Designed for the Prevention of Gluten Cross-Contamination in Food Services

**DOI:** 10.3390/nu10091274

**Published:** 2018-09-10

**Authors:** Priscila Farage, Renata Puppin Zandonadi, Verônica Cortez Ginani, Lenora Gandolfi, Eduardo Yoshio Nakano, Riccardo Pratesi

**Affiliations:** 1 Department of Nutrition, School of Health Sciences, University of Brasilia (UnB), Campus Darcy Ribeiro, Asa Norte, Brasilia DF 70910-900, Brazil; renatapz@yahoo.com.br (R.P.Z.); vcginani@gmail.com (V.C.G.); 2 Faculty of Medicine, University of Brasilia (UnB), Campus Darcy Ribeiro, Asa Norte, Brasilia DF 70910-900, Brazil; lenoragandolfi1@gmail.com (L.G.); pratesiunb@gmail.com (R.P.); 3 Department of Statistics, Central Institute of Sciences, University of Brasilia (UnB), Campus Darcy Ribeiro, Asa Norte, Brasilia DF 70910-900, Brazil; eynakano@gmail.com

**Keywords:** gluten, cross-contamination, food safety, check-list, celiac disease

## Abstract

Gluten cross-contamination in gluten-free food may jeopardize treatment of celiac patients. Considering the deficit of appropriate instruments to enable the implementation of safe production practices for gluten-free food, this study aimed to evaluate the application of a check-list elaborated for gluten cross-contamination prevention in food services. The instrument was applied in 60 Brazilian food services. Interobserver reproducibility and internal consistency of the check-list were tested. A score classification was created for establishments according to the food contamination risk assessment. Subsequent to the application and statistical analysis, the original instrument was reduced to a 30-item check-list. In the reproducibility analysis, none of the 30 items showed significant divergence among the evaluators (*p* > 0.05 in the Cochran Q test). The 30-item version of the check-list presented Kuder–Richardson Formula 20 (KR-20) = 0.771, indicating good internal consistency. The proposed classification score is obtained by adding 1 point for each item with an “adequate” response; therefore, the final score may vary between 0 and 30 points. Establishments with up to 15 points exhibit risk of gluten contamination, while establishments with a score above 16 points exhibit low risk of contamination. The check-list displayed good reproducibility and internal consistency, suggesting that it could be a useful gluten contamination control instrument in food services.

## 1. Introduction

Celiac disease (CD) can be described as an immune-mediated enteropathy triggered by the consumption of gluten in genetically predisposed individuals [1]. This condition affects about 1% of the world population, and the treatment consists of a complete gluten-free diet (GFD). Despite recent advances in the field, a GFD remains until now the only effective means to ensure remission of symptoms, normalization of serum autoantibodies indicative of disease activity, and the healing of intestinal histological architecture [2,3].

Patients with CD experience many difficulties when following the GFD, especially regarding out-of-home eating [4]. Gluten might be present in naturally gluten-free food as a result of cross-contamination caused by many factors, such as shared production areas for gluten-containing and gluten-free food, shared equipment, inadequate procedures by restaurant staff, and insufficient kitchenware sanitization practices, among others [5,6].

This problematic situation may even lead to the necessity to adopt a gluten contamination elimination diet, a modified more restrictive gluten-free diet with the purpose of eliminating any possible sources of gluten cross-contamination, as the presence of gluten traces in food may be sufficient to provoke mucosal and clinical alterations that jeopardize recovery in some diet-adherent non-responsive CD patients [7].

The Codex Alimentarius defines ‘gluten-free foods’ as those with gluten level below 20 ppm (mg/kg) [8]. Although many countries adopt the Codex threshold, most lack a consistent monitoring process to evaluate gluten content in food [9]. Moreover, very few studies have been conducted to evaluate strategies to prevent gluten cross-contamination in food services [6,10,11].

There have been some initiatives for the promotion of safe gluten-free food production, such as the Gluten-Free Certification Program, developed by the Canadian Celiac Association. The program—based on the principles of the Hazard Analysis and Critical Control Points system (HACCP)—relies on a manual, a document comprising the program’s standards and policies and on a check-list organized to evaluate the establishment policy and procedures and determine how they compare with the requirements of the program [12]. Although there are similar programs in other countries, most do not mention the use of specific tools and instruments such as the Canadian check-list that helps implement a gluten cross-contamination control system. There is also practically no record in the scientific literature regarding the creation and evaluation of such instruments.

In Brazil, Farage et al. [11] have developed a check-list for the control of gluten contamination in food services. This check-list was based on Brazilian resolutions that cover hygienic-sanitary aspects of food production and the international standard ISO 22,000, regarding food safety management. Issues addressed in those documents were adapted to contemplate food safety aspects related to the production of gluten-free food. Moreover, the check-list of the Canadian Gluten-Free Certification Program was also used, as it addresses specific topics regarding cross-contamination by gluten. The check-list elaborated by Farage et al. [11] has already been validated concerning content and approved by semantic evaluation using the Delphi method, indicating that it may be an interesting tool that can help implement a gluten cross-contamination prevention system. All data regarding elaboration, content validation, and semantic evaluation of the check-list are published elsewhere, as well as the check-list itself [11].

The check-list proposed by Farage et al. [11] has been elaborated to focus on obtaining gluten content in gluten-free food below the level of 20 ppm, as proposed by the Codex and considered safe for celiac patients [11].

For the development of data collection instruments/questionnaires such as check-lists, the phenomena of interest must be translated into concepts that can be measured, observed, or recorded. Many important aspects should be considered in this process to generate a useful tool capable of obtaining the desired information. Thus, a series of elaboration and evaluation steps must be carefully followed [13].

Two essential requirements of a questionnaire are its validity and reliability [14]. The validity refers to the accuracy of the instrument in measuring what it is proposed to measure. Therefore, an instrument is valid when its construction and applicability allow the true measurement of what one intends to measure [15]. On the other hand, an instrument is reliable when it consistently produces similar results, for example, when different evaluators apply it. In this context, the analysis of reproducibility is useful to determine the reliability of the instrument, and it is related to the agreement of answers using tests with the same instrument applied under the same conditions [14,16].

Reliability may also be determined in terms of internal consistency [17]. Internal consistency refers to the existence of correlation between the different items of the instrument and between each item and the total score of the scale, namely the homogeneity of the instrument [18]. A high degree of internal consistency is important because it is related to the ability of the researcher to interpret the composite score as a reflection of the test items [17].

After the construction and evaluation of an instrument, the selection or development of a method to evaluate the answers is important. In instruments that aim to define a diagnosis or identify a situation, a classification score may be necessary. Scores are widely applied in different areas to estimate the probability of an outcome on a quantitative basis [19]. For the development of a score, statistical methods are often used [20].

The process of construction and evaluation of instruments must address all these issues in order to generate reliable results, both in research and in practical situations.

It is already well established in the literature that the necessity to adopt a life-long strict gluten-free diet is associated with the impairment of celiac patients’ quality of life. Consequently, studies that contribute to the development of strategies that help overcome difficulties related to the diet of these individuals are of great value [21].

In view of the scarcity of studies focusing on the prevention of gluten cross-contamination in food, and the deficit of appropriate instruments to enable the implementation of safe production practices for celiac patients and others who need to adopt the GFD, the primary objective of this study was to evaluate the application of a check-list elaborated for the prevention of gluten cross-contamination in food services under real circumstances for which the instrument was designed. The secondary aims of this study were as follows: (i) to analyze the interobserver reproducibility and internal consistency of the check-list; and (ii) to elaborate an evaluation score for the check-list.

The prevention check-list of gluten cross-contamination proposed by Farage et al., (2017) was tested in real conditions in regular food services from Brazil. The instrument had been previously submitted to content validation and semantic evaluation, and presently other important aspects of the questionnaire were also evaluated, such as interobserver reproducibility and internal consistency. Moreover, the authors are now proposing a response score to classify food services according to the risk of offering gluten-contaminated foods.

## 2. Materials and Methods

### 2.1. Study Design

This is an instrument evaluation study with a quantitative–qualitative approach. The check-list previously elaborated and submitted to content validation and semantic evaluation was applied in Brazilian food services under circumstances in which it was designed for other pertinent evaluations, with the purpose of obtaining a suitable instrument for practical use. Data concerning the instrument application were compared with data regarding the presence or absence of gluten in naturally gluten-free food samples collected from these establishments.

This study has been approved by the Ethics Committee of the School of Health Sciences of the University of Brasília (protocol number: 60987816000000030).

### 2.2. Sample

The sample was composed of regular food services from the city of Brasilia (Brazil) that produce both gluten-containing and naturally gluten-free lunch food. Three databases were used to select these establishments. The Regional Nutrition Council provided a record of restaurants of the city. Cafeteria and canteens from the city’s day care centers, schools, nursing homes, and philanthropic institutions assisted by the food and nutrition national security program Mesa Brasil were also invited to participate in the study. Lastly, a list of the government community restaurants was provided by the State Subsecretariat for Food and Nutrition Security. The invitation to participate in the study was made by telephone and/or e-mail contact. Among 282 establishments obtained through these databases, 60 agreed to participate and consented to the researcher’s visit for the application of the check-list on the site.

It should be clarified that control of gluten cross-contamination in food services is not a regular practice in Brazil. The Brazilian legislation does not require cross-contamination prevention in meals from restaurants and does not set a limit of gluten content for food considered gluten-free as advocated in other countries. In Brazil, food that is naturally gluten-free or produced with gluten-free ingredients can be identified as gluten-free without the need for laboratory analysis or any monitoring process to consistently evaluate gluten content. Therefore, refusal to participate in the study is probably not related to fear of identification of contaminated samples in the establishment as this is not a concern of nutritionists and owners of food services in general. Among reasons to deny participation in the study, most common were overloaded establishment routine and lack of available time to receive the researchers.

### 2.3. Application of the Check-List in Food Services

The check-list was applied in food services during the usual production in lunch hours. The instrument was completed based on direct observation of the staff routine, procedures, and practices within the establishment. Moreover, the nutritionist responsible for the food service and staff was questioned in cases of doubts. In these cases, the researchers avoid asking direct questions that could induce an “adequate” response by employees. Although nutritionists were aware of the general goal of the study, they did not have access to detailed information about the methods applied in order to evaluate the possible risk of cross-contamination in the establishment.

The application of the check-list in food services made it possible to identify difficulties related to the interpretation of its items in real conditions. In this phase, the researchers considered it pertinent to change the previously established standard of answers—“Yes/No/Not applicable”—to “Adequate/Inadequate/Not applicable”, as some items were written in the negative form, which made it hard to understand their content and have the appropriate response according to each case filled in.

It is important to mention that the answer “not applicable” was later translated to “adequate” or “inadequate”, depending on its context, for the correct interpretation of data. For example, item ‘2.1.6’ of the check-list refers to the exclusivity of thermal processing equipment such as fryers for the production of gluten-free food [11]. In that case, whenever this type of equipment was not present in the food service, the answer “not applicable” was converted into “adequate” because the absence of the fryer would mean that it would not be a source of contamination.

Also, this step allowed the verification of relevance of the items in practice, which culminated in the exclusion of the last item of the instrument (item 11.3), because it addressed recall procedures regarding products that are no longer under the organization’s control and are posteriorly identified as contaminated. This issue is not applicable to the reality of food services, where food is served and consumed in a particular moment in time, differently from processed industrial products, which may remain in food markets for a long period of time.

### 2.4. Construction of the Check-List Score

The results obtained by the food services check-list were compared with results of the presence or absence of gluten contamination in food samples from these establishments, through a logistic regression model. The objective of this analysis was to create a score for the classification of establishments according to the answered check-list, by adopting the gold standard to evaluate contamination by gluten—quantification of gluten by the enzyme-linked immunosorbent assay in food samples.

For that purpose, a total of three samples of naturally gluten-free dishes produced at the site visit day were randomly chosen from each food service. The collection of the samples was performed on the same day as the completion of the check-list in order to guarantee equivalent conditions. Samples were analyzed by the Ridascreen Gliadin sandwich R5 enzyme-linked immunosorbent assay (ELISA) R-7001 (R-Biopharm, Darmstadt, Germany), according to the manufacturer’s instructions, at the Interdisciplinary Laboratory of Biosciences (Research Laboratory in Celiac Disease), University of Brasília, Brazil. Samples with gluten content above 20 ppm were considered contaminated, as defined by Codex [8]. Therefore, food services were considered “contaminated” if at least one out of three samples displayed gluten content above 20 ppm.

The ELISA R5 technique was chosen for the analysis because it has been endorsed as the type I method by the Codex Alimentarius and recommended by the Association of Official Analytical Chemists (AOAC) for the detection of gluten in food. This technique is appropriated for raw products as well as processed food, as is the case of meals evaluated in this study.

For the construction of the score, 36 items of the original version of the check-list were excluded, as they disclosed the same responses in all the applications of the instrument: “adequate” or “inadequate”, depending on the item, displaying zero variability. The remaining 48 items were ordered according to their statistical significance (through a logistic regression model) and consistent with the specialist experience (importance of the item according to the researchers). In the end, the 30 most relevant items, according to statistical and researchers’ experience significance, were used to compose the proposed final version of the instrument.

The new version of the check-list along with the classification score can be found in Supplementary Materials.

The final instrument was used to define an adequacy score for the classification of food services. The creation of the score aimed to offer an objective measure to identify the risk of the establishment producing contaminated food. The proposed score is obtained by adding one point for each item with an “adequate” response. Items with an “inadequate” response do not add points to the score. Thus, the adequacy score may vary between 0 and 30 points. In this sense, the higher the score of the food service, the more adequate it is and the lower its risk of gluten contamination in the produced meals.

### 2.5. Analysis of Interobserver Reproducibility and Internal Consistency of the Check-List

For the evaluation of interobserver reproducibility, the checklist was applied by three researchers, independently and on the same day, in 8 out of 60 food services that participated in the study. Reliability (reproducibility) was tested using the Cochran Q test, considering a level of significance of 5%. This step was performed considering the 30 items of the check-list final version.

The analysis of the internal consistency of the check-list was also performed considering the 30 most relevant items that were proposed to compose the final version of the instrument. Internal consistency was determined using the Kuder–Richardson Formula 20 (KR-20), which is used for measures with dichotomous choices [17].

Statistical analysis was performed through the software IBM SPSS—Statistical Package for Social Sciences (FIELD, 2000), version 19.0, and R (R CORE TEAM, 2017). All statistical tests were performed considering a 5% significance level.

## 3. Results

Among the 60 food services that took part in this study, a total of four establishments displayed gluten contamination above 20 ppm in the foods sampled. These results (presence or absence of contamination in the establishment) were used for comparison to the results obtained through the application of the check-list in site.

The check-list’s minimum score was 5 points and the maximum was 25 points, considering all 60 food services. The lower the score, the higher the risk of contamination. A mean score of 16.13 (SD = 4.56) was observed. When we perform the classification of establishments using this score, two errors can occur: false positive (the instrument indicates contamination when it does not exist) or false negative (the instrument does not indicate contamination when it does exist). The cut-off was defined in a way to minimize the probability of a false negative. In this way, the classification criteria were proposed as follows: establishments with up to 15 points exhibit risk of gluten contamination, while establishments with a score greater than or equal to 16 points exhibit low risk of contamination. This cut-off yielded 75% sensitivity and 64% specificity (area under the ROC = 0.589; 95% CI: (0.244; 0.935)).

Table 1 shows the classification of the food services according to the check-list score.

The results presented in Table 1 indicate that if the establishment exhibits a score of up to 15 points, the probability of contamination is 13% (risk of contamination). On the other hand, if the establishment displays a score higher than 15 (16 points or more), the probability of contamination is 2.7% (low risk of contamination).

Items that compose the final version of the check-list are presented in Table 2, along with the frequency of responses observed in the sample of 60 food services.

The reproducibility of the instrument was tested question-by-question considering the answers of three evaluators who applied the instrument in eight food services simultaneously and independently. The concordances of the answers were verified by the Cochran’s Q test. None of the 30 items composing the final instrument showed significant divergence among the evaluators (*p* > 0.05 in the Cochran Q test).

For the determination of internal consistency, the Kuder–Richardson Formula 20 (KR-20) was used. KR-20 ranges from 0 to 1, where 0 is no reliability, and 1 is perfect reliability. In general, values above 0.5 are usually considered reasonable [17]. The 30-item version of the check-list presented KR-20 = 0.771, which indicates good internal consistency.

## 4. Discussion

The most common cause of non-response to the treatment of CD is the failure to comply with the GFD, either voluntarily or unintentionally, as in cases of consumption of contaminated products. The presence of gluten traces in the diet may prevent histological and clinical recovery of celiac patients, even leading to an incorrect diagnosis of refractory CD, which would result in unnecessary use of immunosuppressive therapy [7]. The occurrence of gluten cross-contamination has been identified in processed food [3,22,23,24,25] and meals from food services [26,27] by different studies.

Although the consequences of maintaining gluten in the diet of these patients are well described in the literature, very few studies have proposed practical strategies to prevent cross-contamination of gluten-free products and meals. Farage et al. (2017) developed a check-list for the production of safe gluten-free food in food services that has been validated regarding the content and approved in semantic evaluation [11]. In the present study, the check-list has been tested in Brazilian food services.

A reduction of the original instrument to a 30-item check-list is suggested following the results obtained after its application in food services. Some items were removed considering that they displayed zero response variability amongst the establishments and others were removed after appreciation of their relevance to the outcome of contamination through a logistic regression model and specialists experience.

It is evident that a well-structured questionnaire should consider minimizing the physical or mental effort of the respondent. Consequently, an instrument should contain only essential items to accomplish its purpose. In the pilot evaluation of a questionnaire, the researcher may include a greater number of items that will be judged later in regard to their pertinence and representativeness in the reality for which it is applied. At this moment, attributes related to language and comprehension of the instrument may also be evaluated [28]. The application of the check-list in food services in this study led to modifications concerning its size and scale of responses, as well as the verification of the adequacy of items according to the reality of this type of establishment, which may have improved the instrument.

The reduction of the check-list is advantageous because a shorter instrument is more likely to be used because of its practicability. Moreover, interpretation of the score is simple, which may also encourage its application. In this study, items of the check-list considered important by the judges who participated in the content validation process [11] were not associated with a higher risk of gluten contamination. Therefore, the reduced check-list may be a reasonable tool to prevent contamination. However, the original instrument may also be an option for food services that intend to implement all requirements proposed in the items of the extended version.

According to the Codex and the Association of Official Analytical Chemists (AOAC), the gold standard for the analysis of gluten content in food is the R5 ELISA method [8,9]. However, cost and time required for this analysis may turn a regular monitoring procedure at food services difficult. In this context, the use of the check-list and the classification score would represent a more viable alternative, not as a replacement of laboratory analysis, but as complementation that could be applied more frequently, without the immediate need for samples’ analysis.

In this study sample, none of the food services achieved 100% adequacy in the check-list (30 points in the score). This finding may be explained by poor adherence to good manufacturing practices in Brazil, as demonstrated by the study of Akutsu et al. (2005), which classified establishments in Brasilia according to their fulfillment of essential items from the National Surveillance Agency form regarding good practices of food manufacturing [29]. As previously mentioned, the check-list was developed based on documents regarding hygienic-sanitary control procedures [11]. Thus, food services that do not adhere to basic hygiene requirements will probably display low score of adequacy in the check-list for gluten contamination prevention, showing greater risk in providing contaminated food.

The cutoff proposed for the classification of contamination risk is 16 points. The cut-off was defined in a way to minimize the probability of a false negative, that is, to classify as adequate an establishment that displays a risk of contamination. In this sense, if the food service scores at least 16 points, it will be classified as presenting a low risk of contamination, and the probability of this establishment providing contaminated food (probability of false negative) is 2.7%. This cut-off allowed the check-list to correctly classify 75% of contaminated establishments (sensitivity of 75%) and correctly classify 64% of uncontaminated establishments (specificity of 64%).

Concerning the reproducibility of the check-list, the results showed that none of the 30 items composing the final instrument displayed significant divergence among the evaluators, indicating that the instrument is reproducible. Moreover, analysis of internal consistency using the Kuder–Richardson Formula 20 (KR-20) indicated good internal consistency. Both parameters are used in the evaluation of the reliability of instruments. Reliability is related to the constancy of results obtained when the same object is evaluated, measured, or quantified more than once. Without expected attention to these characteristics, the information collected will not be worthy of credit and significance [14]. Data from this study revealed that the check-list displayed satisfactory results as to reliability.

It is important to mention that the low frequency of contamination among the samples from this study may interfere with the accuracy of results. The use of the 30-items version of the check-list may be a viable tool for the evaluation of food services. However, a larger study with a greater number of food samples would provide more certainty about the information obtained.

As pointed out, the Brazilian legislation does not require cross-contamination prevention in meals from restaurants. Therefore, fear of serving gluten contaminated food is not a concern among nutritionists and owners of regular food services in Brazil. It is likely that staff did not understand the study visits as monitoring assessment and consequently employees probably did not alter their regular practices on the visit day. Moreover, food service staff in Brazil usually display a low level of education and do not have access to information regarding gluten and celiac disease. As demonstrated in the study by Laporte and Zandonadi (2011), chefs from restaurants in Brazil are not aware of sources of gluten in food, nor of procedures that may lead to gluten cross-contamination when preparing gluten-free food [30]. This is why the chances of bias in this sense are probably not high.

Reliability of an instrument may be determined from the reproducibility and internal consistency of it. Thus, the check-list proposed in this study may be considered reliable. Nevertheless, this study has no pretension to exhaust the subject because of its complexity and study limitations imposed by the sample size. However, we expect to have contributed to broaden discussions regarding the control of gluten contamination in food services and serve as a starting point for future research.

## 5. Conclusions

Data from this study resulted in a shorter and easier application version of the original check-list proposed by Farage et al. (2017). The reduction of the instrument is a positive outcome, which was only possible after testing the instrument in real time within food services. Moreover, a score was constructed that enables the classification of food services according to their risk of producing contaminated food. The development of the score was based on the comparison of gluten content from samples collected at food services and data regarding the application of the check-list. The interpretation of the score is simple, which can also encourage the use of the instrument.

Studies such as this one are important to broaden the discussion related to gluten cross-contamination, which may benefit celiac patients and others with gluten-related disorders. Strategies of this type may improve access to safe gluten-free food, contributing to the quality of life of these individuals. Although there are other tools used for gluten-free certification processes in many countries, the authors have not identified any specific instrument that was submitted to a validation process and other evaluations like the ones performed in the present study. Therefore, this research is innovative in this sense.

The check-list evaluated in this study displayed good reproducibility and internal consistency, indicating that it is reliable. Therefore, it may be a useful instrument in a gluten contamination control system in food services.

## Figures and Tables

**Table 1:** Classification of the food services according to the check-list score.

Score	Contaminated	Not Contaminated	Total
Up to 15 points	3 (13%)	20 (87%)	23
16 points or more	1 (2.7%)	36 (97.3%)	37
Total	4	56	60

**Table 2:** Items of the final version of the check-list and frequencies of responses observed in the food services.

Item ^1^	Inadequate Frequency (%)	Adequate Frequency (%)	Cochran Q (*p*)
1.1.1. Floor material that allows easy and proper sanitation (smooth, drained with slope, waterproof).	11 (18.3%)	49 (81.7%)	2.00 (0.368)
1.1.2. Floor in proper conservation (free of defects, cracks, holes, and others).	10 (16.7%)	50 (83.3%)	3.50 (0.174)
1.3.2. Wall in proper conservation (free from cracks and peeling).	5 (8.3%)	55 (91.7%)	2.80 (0.247)
1.4.1. Smooth surface doors, adjusted to the jambs, and without coating faults in order to reduce the risk of contamination coming from the external area.	20 (33.3%)	40 (66.7%)	2.00 (0.368)
1.6.1. In case of ramps and workbenches used to support both gluten-free and gluten-containing food, a hygienic procedure is performed between the use of this surface for gluten-containing and gluten-free food.	47 (78.3%)	13 (21.7%)	2.00 (0.368)
1.7.1. Toilets equipped with washbasins and products intended for personal hygiene: antiseptic odorless liquid soap or odorless liquid soap and antiseptic, non-recycled paper towel or other safe and hygienic drying system, collectors with lid and without manual activation.	22 (36.7%)	38 (63.3%)	4.00 (0.135)
1.8.1. Existence of washbasins in the production area with running water, in appropriate positions in relation to the production and service flow, with sufficient number to suit the entire production area, preferably equipped with automatic stopcock, antiseptic odorless liquid soap or odorless liquid soap and antiseptic, non-recycled paper towels or other hygienic and safe drying system, and paper collectors without manual activation.	26 (43.3%)	34 (56.7%)	4.50 (0.105)
1.9.1. Artificially air-conditioned environments, without fans, without generating airflow and absence of natural airflow from the production area of gluten-containing food to the production area of gluten-free food, avoiding an environment with particles in suspension.	17 (28.3%)	43 (71.7%)	2.00 (0.368)
1.10.1. Facilities kept under appropriate hygienic-sanitary conditions, that is, without the presence of accumulation of residues, with proof by means of registration in specific spreadsheets, updated and with information consistent with what is being observed.	31 (51.7%)	29 (48.3%)	0.33 (0.846)
1.10.2. Utensils used for the cleaning of facilities distinct from those used for the cleaning of equipment that come into contact with food, with hygiene products and utensils exclusive for the use in the production area of gluten-free food.	58 (96.7%)	2 (3.3%)	2.00 (0.368)
1.11.1. Containers for the collection of waste inside the establishment that are easily sanitized (i.e., without cracks that allow dirt to accumulate and are difficult to access by cleaning utensils) and transported (i.e., can be easily moved by those responsible for the procedure); emptied whenever its content reaches 2/3 of its capacity and constantly sanitized, showing no evidence of accumulated dirt; use of appropriate garbage bags.	9 (15.0%)	51 (85.0%)	3.15 (0.174)
1.11.2. Waste removed from the gluten-containing food production area does not pass through the production area of gluten-free food.	31 (51.7%)	29 (48.3%)	1.50 (0.472)
1.12.1. Layout suitable for the productive process: number, capacity and distribution of dependencies according to the branch of activity, production volume, and expedition.	6 (10.0%)	54 (90.0%)	0.00 (1.000)
1.12.2. Areas for receiving and depositing ingredients distinct from the areas of production, storage, and expedition of the final product.	11 (18.3%)	49 (81.7%)	1.00 (0.607)
2.1.3. Production line equipment (mixers, processors, blenders, toasters, etc.) identified and exclusive to the production of gluten-free food.	28 (46.7%)	32 (53.3%)	2.00 (0.367)
2.1.4. Food preservation equipment (refrigerators, freezers, cold rooms) exclusive for gluten-free products or, when not possible, the disposal of products is done in separate spots and/or with some kind of physical separation between gluten-free and gluten-containing products.	33 (55.0%)	27 (45.0%)	0.00 (1.000)
2.1.5. Thermal processing equipment (ovens) exclusive for gluten-free food or, when of common use, not used for baking gluten-free and gluten-containing food simultaneously.	11 (18.3%)	49 (81.7%)	1.00 (0.607)
2.1.6. Thermal processing equipment (fryers, hot plate for tapiocas, pancakes, and others) exclusive for gluten-free food.	18 (30.0%)	42 (70.0%)	1.00 (0.607)
3.1.1. Employees display proper personal cleanliness: body cleanliness, clean hands, short nails, clean uniforms.	10 (16.7%)	50 (83.3%)	1.00 (0.607)
3.1.2. Employees use a uniform exclusive for handling gluten-free food or a uniform that has not been previously used to handle food with gluten, without having been washed afterwards.	51 (85.0%)	9 (15.0%)	0.00 (1.000)
3.2.1. There is guidance (posters) for proper hand hygiene, which includes appropriate moments and procedures, accessible to employees and followed correctly.	41 (68.3%)	19 (31.7%)	1.00 (0.607)
3.2.2. Employees do not handle gluten-containing and gluten-free foods simultaneously or engage in any act that could lead to cross-contamination, such as eating during food preparation.	48 (80.0%)	12 (20.0%)	0.00 (1.000)
4.1.2. Defrosting of gluten-free food held in a separate location from gluten-containing food and without getting in touch with utensils and equipment where gluten-containing food is stored or held in locations that are cleaned before procedure.	16 (26.7%)	44 (73.3%)	2.00 (0.368)
4.2.2. Water or oil previously used in the preparation of gluten-containing food is not reused at the preparation of gluten-free food.	12 (20.0%)	48 (80.0%)	1.14 (0.565)
4.3.2. Segregation or separation of procedures such as production scheduling or specific/exclusive lines for gluten-free food, with an ordered flow without crossing between gluten-free and gluten-containing food.	49 (81.7%)	11 (18.3%)	0.00 (1.000)
4.4.2. Labeling statements with visible identification and in accordance with current legislation regarding the presence or absence of gluten.	45 (75.0%)	15 (25.0%)	0.00 (1.000)
5.1. At the distribution of food, employees follow procedures to eliminate the risk of gluten contamination, through hand hygiene, use of protective utensils and disposable gloves, and others whenever there is previous contact with gluten-containing food.	59 (98.3%)	1 (1.7%)	0.00 (1.000)
5.3. Preparation identified with labels or other visible method according to its gluten content.	41 (68.3%)	19 (31.7%)	0.33 (0.846)
5.5. Monitoring of the preparation identification plates in regards to the presence/absence of gluten at the moment of distribution.	46 (76.7%)	14 (23.3%)	1.20 (0.549)
6.1.1. Operations carried out at the facility are in accordance with an on-site Good Practices Manual that meets the legal requirements in regards to content and updating.	20 (33.3%)	40 (66.7%)	0.50 (0.779)

^1^ The original numbering of the items was maintained in Table 1 to facilitate their identification in regard to the previous version of the check-list [11].

## Supplementary Materials

The following is available online at http://www.mdpi.com/2072-6643/10/9/1274/s1, File S1: Check-list for the verification of non-conformities related to gluten-contamination in food services.
