# Supplementary material for: Gluten-Free Diet: From Development to Assessment of a Check-List Designed for the Prevention of Gluten Cross-Contamination in Food Services
Source: Nutrients. 2018 Sep 10;10(9):1274. doi: 10.3390/nu10091274 (PMC6165388; doi:10.3390/nu10091274)
Supplement: Supplementary file 1 [file nutrients-10-01274-s001.pdf]

nutrients

Article

Gluten-Free Diet: From Development to Assessment of a Check-List Designed for the Prevention of Gluten Cross-Contamination in Food Services

Priscila Farage [1,*], Renata Puppin Zandonadi [1], Verônica Cortez Ginani [1], Lenora Gandolfi [2], Eduardo Yoshio Nakano [3], and Riccardo Pratesi [2]

[1] Department of Nutrition, School of Health Sciences, University of Brasilia (UnB), Campus Darcy Ribeiro, Asa Norte, Brasilia DF 70910-900, Brazil; renatapz@yahoo.com.br (R.P.Z.); vcginani@gmail.com (V.C.G.)
[2] Faculty of Medicine, University of Brasilia (UnB), Campus Darcy Ribeiro, Asa Norte, Brasilia DF 70910-900, Brazil; lenoragandolfi1@gmail.com (L.G.); pratesiunb@gmail.com (R.P.)
[3] Department of Statistics, Central Institute of Sciences, University of Brasilia (UnB), Campus Darcy Ribeiro, Asa Norte, Brasilia DF 70910-900, Brazil; eynakano@gmail.com
* Correspondence: pri_farage@hotmail.com; Tel.: +55-61-9818-70144

Supplementary Materials

Check-list for the verification of non-conformities related to gluten-contamination in food services

**Legend:**			
**A – Adequate**	**I – Inadequate**	**NA – Not applicable**	**OBS – Observation**

**Number:**			**Year:**
**Company identification:**			
**Company name:**			
**Trading name:**			
**Health license:**	**State / Municipal registration:**		
**National record of legalized person/ individual registration:**	**Phone:**	**Fax:**	
**E-mail:**			
**Adress:**			
**Neighborhood:**	**City:**	**State:**	**Zip code:**
**Activity branch:**		**Monthly output:**	
**Number of employees:**		**Number of shifts:**	
**Products' categories:**			
**Category description:**			
**Technical manager:**	**Academic background of the technical manager:**		

**Is there an employee responsible for the good manufacturing practices in the establishment?**<br>**( ) Yes ( ) No**	**Academic background of the employee responsible for the good manufacturing practices:**<br>**( ) Training course**<br>**( ) Technical course. Which?**<br>**( ) College degree. On what?**
**Legal representative/owner of the establishment:**	

ITEMS	A	I	NA	OBS
**1. Building and facilities**				
**1.1. Floor**				
1.1.1. Floor material that allows easy and proper sanitation (smooth, drained with slope, waterproof).				
1.1.2. Floor in proper conservation (free of defects, cracks, holes, and others).				
**1.3. Walls**				
1.3.2. Wall in proper conservation (free from cracks and peeling).				
**1.4. Doors**				
1.4.1. Smooth surface doors, adjusted to the jambs and without coating faults in order to reduce the risk of contamination coming from the external area.				
**1.6. Stairs, service elevators, goods lift, and auxiliary structures**				
1.6.1. In case of ramps and workbenches used to support both gluten-free and gluten-containing food, a hygienic procedure is performed between the use of this surface for gluten-containing and gluten-free food.				
**1.7. Toilets and dressing rooms for employees**				
1.7.1. Toilets equipped with washbasins and products intended for personal hygiene: antiseptic odorless liquid soap or odorless liquid soap and antiseptic, non-recycled paper towel or other safe and hygienic drying system, collectors with lid and without manual activation.				

**1.8. Washbasins in the production area**				
1.8.1. Existence of washbasins in the production area with running water, in appropriate positions in relation to the production and service flow, with sufficient number to suit the entire production area, preferably equipped with automatic stopcock, antiseptic odorless liquid soap or odorless liquid soap and antiseptic, non-recycled paper towels or other hygienic and safe drying system and paper collectors without manual activation.				
**1.9. Ventilation and air conditioning**				
1.9.1. Artificially air-conditioned environments, without fans, without generating airflow and absence of natural airflow from the production area of gluten-containing food to the production area of gluten-free food, avoiding an environment with particles in suspension.				
**1.10. Cleaning of the facilities**				
1.10.1. Facilities kept under appropriate hygienic-sanitary conditions, that is, without the presence of accumulation of residues, with proof by means of registration in specific spreadsheets, updated and with information consistent with what is being observed.				
1.10.2. Utensils used for the cleaning of facilities distinct from those used for the cleaning of equipment that come into contact with food, with hygiene products and utensils exclusive for the use in the production area of gluten-free food.				
**1.11. Waste management**				
1.11.1. Containers for the collection of waste inside the establishment which are easily sanitized (ie without cracks that allow dirt to accumulate and are difficult to access by cleaning utensils)				

and transported (ie can be easily moved by those responsible for the procedure); emptied whenever its content reaches 2/3 of its capacity and constantly sanitized, showing no evidence of accumulated dirt; use of appropriate garbage bags.				
1.11.2. Waste removed from the gluten-containing food production area does not pass through the production area of gluten-free food.				
**1.12. Layout**				
1.12.1. Layout suitable for the productive process: number, capacity and distribution of dependencies according to the branch of activity, production volume and expedition.				
1.12.2. Areas for receiving and depositing ingredients distinct from the areas of production, storage and expedition of the final product.				
**2. Equipment, furniture and kitchenware**				
**2.1. Equipment**				
2.1.3. Production line equipment (mixers, processors, blenders, toasters, etc.) identified and exclusive to the production of gluten-free food.				
2.1.4. Food preservation equipment (refrigerators, freezers, cold rooms) exclusive for gluten-free products or, when not possible, the disposal of products is done in separate spots and / or with some kind of physical separation between gluten-free and gluten-containing products.				
2.1.5. Thermal processing equipment (ovens) exclusive for gluten-free food or, when of common use, not used for baking gluten-free and gluten-containing food simultaneously.				
2.1.6. Thermal processing equipment (fryers, hot plate for tapiocas, pancakes				

and others) exclusive for gluten-free food.				
**3. Food service employees**				
**3.1. Clothing**				
3.1.1. Employees display proper personal cleanliness: body cleanliness, clean hands, short nails, clean uniforms.				
3.1.2. Employees use a uniform exclusive for handling gluten-free food or a uniform which has not been previously used to handle food with gluten, without having been washed afterwards.				
**3.2. Hygienic habits**				
3.2.1. There is guidance (posters) for proper hand hygiene, which includes appropriate moments and procedures, accessible to employees and followed correctly.				
3.2.2. Employees do not handle gluten-containing and gluten-free foods simultaneously or engage in any act that could lead to cross-contamination, such as eating during food preparation.				
**4. Food production and transport**				
**4.1. Raw materials, ingredients and package**				
4.1.2. Defrosting of gluten-free food held in a separate location from gluten-containing food and without getting in touch with utensils and equipment where gluten-containing food is stored or held in locations that are cleaned before procedure.				
**4.2. Selection of recipes and ingredients and food preparation**				
4.2.2. Water or oil previously used in the preparation of gluten-containing food is not reused at the preparation of gluten-free food.				
**4.3. Production flow**				

4.3.2. Segregation or separation of procedures such as production scheduling or specific/exclusive lines for gluten-free food, with an ordered flow without crossing between gluten-free and gluten-containing food.				
**4.4. Labeling and storage of final product and/or semi-prepared products**				
4.4.2. Labeling statements with visible identification and in accordance with current legislation regarding the presence or absence of gluten.				
**5. Distribution**				
5.1. At the distribution of food, employees follow procedures to eliminate the risk of gluten contamination, through hand hygiene, use of protective utensils and disposable gloves and others whenever there is previous contact with gluten-containing food.				
5.3. Preparation identified with labels or other visible method according to its gluten content.				
5.5. Monitoring of the preparation identification plates in regards to the presence / absence of gluten at the moment of distribution.				
**6. Documentation**				
**6.1. Manual of good practices**				
6.1.1. Operations carried out at the facility are in accordance with an on-site Good Practices Manual that meets the legal requirements in regards to content and updating.				

FINAL SCORE	**Adequate**	**Inadequate**
**Instructions:** Add one point for each item with an "adequate" response. Items with an "inadequate" response do not add points to the score. For the interpretation of results, “not applicable” answers must be converted to “adequate” or “inadequate”, according to the		

situation (adjudicate whether in that case the "not applicable" represents a risk of food contamination or not).		
**Classification:**<br>- **Establishments ≤ 15 points:** risk of gluten contamination.<br>- **Establishments ≥16 points:** low risk of contamination.	**Total of points:**	
